# Systems aging clock: A novel epigenetic aging clock modeled from organ & bodily function based mortality indices

**DOI:** 10.1093/geroni/igab046.3736

**Published:** 2021-12-17

**Authors:** Raghav Sehgal, Morgan Levine

**Affiliations:** Yale University, New Haven, Connecticut, United States

## Abstract

A diverse array of aging clocks, derived from a variety of omics data and clinical biomarkers, have been developed to describe aging and predict age-related disease. As such, these biomarkers are particularly applicable for use in observational studies, basic science and clinical trials focused on tackling biological aging. However, ongoing research suggests significant heterogeneity in aging, with deterioration and disease occurring in different organ systems or functional domains at various rates across individuals. Existing aging clocks only measure heterogeneity in the degree of aging, not in the manner of aging (e.g. different organ systems or functional domains). We hypothesize these unique trajectories exist and that they can be captured using a systems based approach. In our work, using clinical chemistry biomarkers from participants in the Health and Retirement Study (HRS), Framingham Heart study (FHS) and Women’s Health Initiative (WHI) , we modeled unique epigenetic aging trajectories from distinct groups of biological processes (such as Immune function, metabolic function, hepatic function, cardiac function, renal function and more). Interestingly, these biological system specific scores when combined gave an aging clock with superior mortality prediction than any published aging clock. We further validate the system aging scores and aging clock in different clinical studies to show the added advantage of such a measure, such as the fact that people with similar epigenetic age may have very different system scores. Overall, this method introduces the potential for quantitative and multi-dimensional, personalized aging scores that are indicative of an individual’s disease and disorder risk.

